# Participation in sport and physical activity: associations with socio-economic status and geographical remoteness

**DOI:** 10.1186/s12889-015-1796-0

**Published:** 2015-04-29

**Authors:** Rochelle M Eime, Melanie J Charity, Jack T Harvey, Warren R Payne

**Affiliations:** Institute of Sport, Exercise and Active Living, Victoria University, Melbourne, Australia; School of Health Sciences and Psychology, Federation University, Ballarat, Australia

**Keywords:** Sport, Physical activity, Socio-economic status, Rurality

## Abstract

**Background:**

Many factors influence participation in sport and Physical Activity (PA). It is well established that socio-economic status (SES) is a critical factor. There is also growing evidence that there are differences in participation patterns according to residential location. However, little is known more specifically about the relationship of PA participation and frequency of participation in particular contexts, to SES and residential location. This study investigated the relationship of participation, and frequency and context of participation, to SES and location.

**Methods:**

Three aspects of participation were investigated from data collected in the Exercise, Recreation and Sport Survey (ERASS) 2010 of persons aged 15+ years: any participation (yes, no), regular participation (<12 times per year, ≥12 times per year) and level of organisation of participation setting (non-organised, organised non-club setting, club setting).

**Results:**

The rates of both any and regular PA participation increased as SES increased and decreased as remoteness increased. However, participation in PA was SES- or remoteness-prohibitive for only a few types of PA. As remoteness increased and SES decreased, participation in many team sports actually increased. For both SES and remoteness, there were more significant associations with overall participation, than with regular participation or participation in more organised contexts.

**Conclusions:**

This study demonstrates the complexity of the associations between SES and location across different contexts of participation. Nevertheless, it seems that once initial engagement in PA is established, SES and remoteness are not critical determinants of the depth of engagement.

**Electronic supplementary material:**

The online version of this article (doi:10.1186/s12889-015-1796-0) contains supplementary material, which is available to authorized users.

## Background

There is an abundance of knowledge of the wide range of influences on participation in physical activity (PA). In accordance with the Socio-Ecological model, these influences or determinants of participation can relate to intrapersonal, interpersonal, organisational, environmental, and policy factors [1,2].

One key influence on participation is Socio-Economic Status (SES). This determinant impacts upon many PA determinants across a number of the Socio-Ecological model’s domains [3]. It is consistently reported in both quantitative and qualitative studies that people with higher SES are more likely than those with lower SES to participate in PA, and more specifically in sport [4-7].

A qualitative study of adults in the Netherlands, USA and Republic of Korea found that some barriers to PA and sport participation were consistently reported across all three countries. Along with time pressure, cost was articulated consistently throughout as a barrier to PA participation [8]. In addition to individual and household SES, there is evidence that neighbourhood SES is also related to PA participation. There is evidence that higher SES neighbourhoods have significantly more PA facilities than lower SES neighbourhoods, thus providing more opportunities to be physically active [9]. Furthermore, low SES neighbourhoods were found to have significantly fewer free-for-use facilities than high SES neighbourhoods [9].

There are also differences amongst participation levels and trends according to different geographical regions [10-12]. It is not uncommon for studies to report PA differences according to residence in metropolitan or regional/rural locations [11,12]. There are also reports of variations of PA levels within state capital cities [10] and between different regional communities [11].

Many studies that do report PA according to different geographical regions, use very broad definitions, for example northern and southern regions of a country [6]. While specific measures of location or remoteness exist, these have rarely been used in research in this area. ARIA+ is a geographical measure of remoteness for Australia [13]. A study that adopted this measure of remoteness investigated PA levels amongst adolescents [14]. Both males and females living in major cities reported significantly lower moderate and vigorous PA (MVPA) minutes than males and females living in any other type of region. Participation in sport, however did not differ across regional classifications [14].

In terms of health-enhancing PA, frequency of participation is a key component. It is also important to understand the context of participation. Some studies incorporate frequency as a measure, especially when categorising individuals as meeting or failing to meet the recommended or health-enhancing levels of PA [10]. One important aspect of the context of leisure-time PA has been termed ‘mode’ [15], the four modes being: team sport, individual sport, organised but non-competitive PA, and non-organised PA [15]. There are likely to be differences in participation trends across these modes, however little attention has been paid to specific modes beyond the study of adolescents by Eime and colleagues [15].

In summary, many factors influence participation in sport and PA. It is well established that SES is a critical factor. There is also growing evidence that there are differences in participation patterns according to residential location. However, little is known more specifically about the relationship of PA participation, and frequency of participation in particular contexts, to SES and residential location.

This study investigates the association of participation, and regularity and organisational context of participation, with SES and location.

## Methods

Data collected in the Exercise, Recreation and Sport Survey (ERASS), 2010 was obtained. The usefulness of the ERASS survey from a public health perspective has been established [10,16]. Importantly, it is useful as a national surveillance of habitual PA behaviours and specifically identifies the types of activities undertaken [16]. It has also been used to determine adult participation trends in Leisure Time Physical Activity (LTPA) according to city of residence [10].

Quarterly survey samples for ERASS were selected from all persons aged 15 years and over, living in occupied private dwellings using Computer-assisted Telephone Interviewing. In each quarter approximately 3,400 persons were sampled Australia-wide from all states and territories. Verbal informed consent was indicated by the respondents’ willingness to participate in the telephone survey. De-identified data from the 2010 survey period were analysed in this investigation. Ethics approval was granted by the University Human Research Ethics Committee.

Respondents were first asked whether they participated in any PA during the 12 months prior to the survey. Those who had done so were asked to nominate up to 10 types of PA from a classification of 95 types (e.g. basketball, tennis, aerobics, walking), including both sports (defined as a physical activity that by its nature has a sport governing body and by its nature and organisation, is competitive and is generally accepted as being a sport)[17] and other forms of recreational PA. Hence, for each of the 95 ERASS PA types, each respondent was classified as a participant or a non-participant. For participants in each PA type, two further aspects of participation were investigated: frequency of participation in the 12 months prior to the survey and level of organisation of participation setting (non-organised, organised non-club setting, club setting). After consultation with sport governing bodies, regular participation was defined as at least 12 times in the 12 months prior to the survey, i.e. at least monthly on average. With regard to level of organisation, a person can engage in a particular type of PA in more than one setting. In accordance with the hierarchical precedence of participation settings articulated by Eime et al. [18] all persons who participated in a club setting were classified as club participants, regardless of whether they also participated in other settings. Of those remaining, persons who participated in an organised non-club setting were classified as organised non-club participants, regardless of whether they also participated in non-organised settings. Those remaining participated in only non-organised settings, and were classified as such.

Thus, the following PA indicators (outcome variables) were defined for each respondent: participation in any type of PA (yes/no), participation in each of 95 types of PA (yes/no), regular participation in up to 10 types of PA (yes: ≥12 times per year, no: <12 times per year), and level of organisation of participation in up to 10 types of PA (non-organised, organised non-club setting, club setting).

Socio-economic status was represented by the Australian Bureau of Statistics (ABS) Socio-economic Indices for Areas (SEIFA) Index of Relative Socio-economic Advantage and Disadvantage (IRSAD) [19].The SEIFA IRSAD value assigned to each respondent was the 2011 SEIFA value assigned by ABS to the residential postcode of the respondent [19]. For ease of interpretation, analysis was based on ERASS quintiles of SEIFA IRSAD. SEIFA IRSAD scores are centred on 1000, with a range in the 2010 ERASS sample from 619.55 to 1164.41. The four quintile cutoffs for the 2010 ERASS sample were 938.80, 979.77, 1019.46 and 1065.35.

Access to services and remoteness was represented by five standard categories based on the Access and Remoteness Index for Australia (ARIA+) [13]. These categories are: Major cities, Inner regional, Outer regional, Remote and Very remote. The ARIA+ category assigned to each respondent was the 2011 ARIA+ category assigned to the residential postcode of the respondent. Because the sample sizes in the two most remote of the five ARIA+ categories were small (see Table 1), the ARIA+ measure was collapsed into three categories: major cities, inner regional, and other (i.e. outer regional, remote, very remote).

**Table 1:** **- Respondent Characteristics**

	**All respondents**		**PA participants**	
	**n**	**%**	**n**	**%**
SEIFA IRSAD quintile/range (of residential postal area)	21,593		17,435	
1 (619.55-938.8)	4,332	20.1	3,267	18.7
2 (938.81-979.77)	4,306	19.9	3,330	19.1
3 (979.79-1019.46)	4,334	20.1	3,473	19.9
4 (1019.48-1065.35)	4,326	20.0	3,623	20.8
5 (1065.51-1164.41)	4,295	19.9	3,742	21.5
ARIA+ category/range (of residential postal area)	21,603		17,445	
Major cities of Australia (0–0.20)	11,257	52.1	9,320	53.4
Inner regional Australia (>0.20 - 2.40)	5,103	23.6	4,019	23.0
Outer regional Australia (>2.40 - 5.92)	4,290	19.9	3,352	19.2
Remote Australia (>5.92 - 10.53)	690	3.2	541	3.1
Very remote Australia (>10.53)	263	1.2	213	1.2
Gender	21,603		17,445	
Male	9,452	43.8	7,719	44.2
Female	12,151	56.2	9,726	55.8
Age	Mean	Range	Mean	Range
	49.9	15-98	48.6	15-96

All analyses used ERASS data weighted at the state, region (metropolitan, rest of state), age group and gender levels. Analyses were conducted using SPSS Version 21. Analyses were conducted of the relationship of each of the three outcome variables with the two predictors (SEIFA quintile and ARIA category).

For the two dichotomous outcome variables (participation, regular participation), binary logistic regression was used to investigate the relationship with each of the two predictors. The results were expressed in terms of rates: the participation rate, estimated by the proportion of the sample who reported participating, and the rate of regular participation, estimated by the proportion of participants who were regular participants. Any significant relationship was further investigated to determine the nature of that relationship. The method of polynomial contrasts was used to break down the (frequently curvilinear) relationship between the log odds of the outcome and the predictor into a linear component and any second-, third- or fourth-order components, each of which was independently assessed for statistical significance. For reporting purposes, the relationships identified were classified as positive linear, negative linear or non-linear. Positive and negative linear relationships were defined by the sign of the log-odds value. A non-linear relationship was generally a second-, third- or fourth- order relationship, possibly superimposed on a linear trend. However, in a few cases an overall statistically significant relationship was shown but this could not be characterised into a polynomial pattern.

For the ordinal outcome variable (level of organisation), crosstabulation analysis was conducted, and Goodman and Kruskal’s gamma, designed to measure the concordance of two ordinal variables known to have tied observations, was used to identify any statistically significant association between each predictor and the level of organisation. For reporting purposes, a positive relationship was defined as a positive value of Goodman and Kruskal’s gamma between predictor and outcome variable, while a negative relationship was defined as a negative value of Goodman and Kruskal’s gamma.

## Results

Table 1 summarises the gender, age, SEIFA IRSD and ARIA+ profiles of: 1) all ERASS 2010 survey respondents; and 2) those respondents who reported participating in recreational PA in the twelve months prior to the survey. Table 2 shows the nature of the relationship between each of the three PA participation indicators, with PA aggregated across all 95 types of PA, and the two predictors. For this aggregated analysis, a participant was classified as a regular or non-regular participant on the basis of the highest frequency they reported across all of their (up to 10) reported PA types. Similarly, the level of organisation was assigned to each participant on the basis of the highest level of organisation reported across all of their reported PA types. Table 2 shows that the rates of both PA participation in general and regular PA participation increased as SES (SEIFA IRSD quintile) increased and decreased as remoteness (ARIA+ category) increased. In the case of SEIFA IRSAD, there was a linear trend with some non-linearity superimposed. Conversely, the level of organisation of PA context increased as remoteness increased, and decreased as SES increased.

**Table 2:** **- Relationships between three PA participation indicators and two predictors**

			**Relationship**			
**Indicator**	**Predictor**	**n**	**+ve linear**	**-ve linear**	**Non- linear** ^**1**^	**p-value** ^**2**^
Rate of any participation (All respondents)	SEIFA IRSAD quintile	21,596	*		*	<0.001
	ARIA+ category	21,603		*		<0.001
Rate of regular participation (Participants only)	SEIFA IRSAD quintile	17,733	*		*	<0.001
	ARIA+ category	17,739		*		<0.001
			+ve^3^	-ve^3^		p-value^4^
Level of organisation of participation setting^5^	SEIFA IRSAD quintile	17,421		*		
	ARIA+ category	17,425	*			<0.001

^1^Includes any significant 2nd, 3rd or 4th order relationship.

^2^Logistic regression likelihood ratio test.

^3^ Based on the sign of Goodman and Kruskal’s gamma coefficient.

^4^p-value for Goodman and Kruskal’s gamma coefficient.

^5^1 = unorganised, 2 = organised non-club, 3 = club

The subsequent tables summarise the nature of the relationship between the two predictors and each of the three PA participation indicators, separately for each of the 95 types of PA.

Table 3 shows the nature of the relationship between the rate of any participation in PA and SEIFA quintile. Forty two of the 95 types of PA were shown to have a statistically significant relationship between rate of participation and SEIFA IRSAD quintile. Twenty five had a positive linear relationship between rate of participation and SEIFA IRSAD quintile, with 16 of these also having a superimposed non-linear relationship. Ten had a negative linear relationship between rate of participation and SEIFA IRSAD quintile, of which seven also had a superimposed non-linear relationship. Five had a solely non-linear relationship between participation and SEIFA IRSAD quintile, with no significant linear trend. Additional file 1: Table S3A provides detailed examples illustrating different patterns of relationship. It should be borne in mind that the term “linear” applies to the relationship between the log odds of participation (not the odds of participation) and the SEIFA quintile.

**Table 3:** **- Relationship between rate of participation in particular types of physical activity and quintiles of SEIFA IRSAD**

		**Relationship with SEIFA IRSAD quintile**			
**Physical activity** ^**1**^	**n**	**Positive linear**	**Negative linear**	**Non- linear** ^**2**^	**p-value** ^**3**^
Aerobics/fitness	21,597	*		*	<0.001
Australian rules football	21,598			*	<0.001
Badminton	21,597	*			0.019
Basketball	21,599	*			0.015
Boxing	21,598	*		*	<0.001
Bush walking	21,597	*		*	<0.001
Canoeing/kayaking	21,598	*		*	0.002
Cricket (indoor)	21,597			*	0.001
Cricket (outdoor)	21,598		*	*	<0.001
Cycling	21,597	*		*	<0.001
Fishing	21,597		*	*	<0.001
Football (indoor)	21,598	*		*	<0.001
Football (outdoor)	21,597	*		*	<0.001
Golf	21,597	*		*	<0.001
Gridiron	21,598			*	<0.001
Horse riding/equestrian/polo cross	21,598		*		0.013
Ice/snow sports	21,599	*			<0.001
Lawn bowls	21,598		*		<0.001
Motor sports	21,597		*	*	<0.001
Netball	21,597			*	0.018
Orienteering	21,597	*			0.015
Rock climbing	21,598	*			<0.001
Roller sports	21,599	*			<0.001
Rowing	21,597	*			<0.001
Rugby league	21,598		*	*	<0.001
Rugby union	21,597	*		*	<0.001
Running	21,598	*		*	<0.001
Sailing	21,597	*		*	<0.001
Shooting sports	21,596		*	*	<0.001
Surf sports	21,598	*		*	<0.001
Swimming	21,597	*			<0.001
Table tennis	21,597		*		0.030
Tennis	21,599	*		*	<0.001
Tenpin bowling	21,597		*	*	0.005
Touch football	21,597		*	*	<0.001
Triathlons	21,598	*		*	0.001
Volleyball	21,597			*	<0.001
Walking	21,598	*			<0.001
Water polo	21,598				<0.001
Water-skiing/power boating	21,597				0.037
Weight training	21,598	*		*	0.013
Yoga	21,598	*		*	<0.001

^1^For the remaining 53 types of physical activity, there was no significant relationship between SEIFA IRSAD quintile and the rate of regular participation.

^2^Includes any significant 2nd, 3rd or 4th order relationship.

^3^Logistic regression likelihood ratio test.

Table 4 shows the nature of the relationship between rate of regular participation and SEIFA IRSAD quintile. Twenty one of the 95 types of PA were shown to have a significant relationship between rate of regular participation and SEIFA IRSAD quintile. Of these, one had a ‘purely’ positive linear relationship, three had a ‘purely’ negative linear relationship, and two had a negative but non-linear relationship. The remaining 13 had non-linear relationships with no linear component. Additional file 1: Table S4A provides detailed examples illustrating different patterns of relationship.

**Table 4:** **- Relationship between rate of regular** ^**1**^ **participation in particular types of physical activity and quintiles of SEIFA IRSAD**

		**Relationship with SEIFA IRSAD quintile**			
**Physical activity** ^**2**^	**n**	**Positive linear**	**Negative linear**	**Non-linear** ^**3**^	**p-value** ^**4**^
Aquarobics	229			*	0.017
Athletics/track and field	144	*			<0.001
Australian rules football	712		*		0.018
Basketball	746	*		*	0.002
Boxing	254			*	0.047
Bush walking	1,038		*		0.024
Cricket (outdoor)	694			*	0.038
Football (indoor)	412			*	<0.001
Football (outdoor)	1,036			*	<0.001
Lawn bowls	441		*	*	0.046
Martial arts	450			*	0.032
Rugby league	292			*	0.001
Scuba diving	118			*	0.020
Shooting sports	164		*	*	<0.001
Swimming	2,797				0.035
Table tennis	119			*	0.010
Tenpin bowling	130			*	0.011
Touch football	597			*	<0.001
Walking	7,716			*	0.001
Water-skiing/power boating	142		*		0.017
Weight training	632			*	0.006

^1^Twelve times or more v fewer than 12 times in past 12 months.

^2^For the remaining 74 types of physical activity, either there was no significant relationship between SEIFA IRSAD quintile and the rate of regular participation, or the sample size was too small for valid statistical analysis.

^3^Includes any significant 2nd, 3rd or 4th order relationship.

^4^Logistic regression likelihood ratio test.

Table 5 shows the nature of the relationship between level of organisation of participation and SEIFA IRSAD quintile. Because level of organisational participation is an ordinal variable (as opposed to a quantitative rate associated with a binary variable), the issue of linearity does not apply. Twenty of the 95 types of PA were shown to have a significant relationship (concordance) between level of organisation of PA and SEIFA IRSAD quintile. Six concordances were positive and 14 were negative.

**Table 5:** **Relationship between level of organisation** ^**1**^ **of particular types of physical activity and quintiles of SEIFA IRSAD**

		**Relationship with SEIFA IRSAD quintile**		
**Physical activity** ^**2**^	**n**	**Positive**	**Negative**	**p-value** ^**3**^
Air sports	17		*	<0.001
Athletics/track and field	142	*		<0.001
Australian rules football	686		*	<0.001
Basketball	746		*	0.008
Billards/snooker/pool	16		*	<0.001
Canoeing/kayaking	267	*		0.021
Cricket (indoor)	147		*	0.007
Cycling	2,491	*		0.002
Football (outdoor)	1,009		*	<0.001
Golf	1,408		*	<0.001
Hockey (indoor)	32	*		0.001
Hockey (outdoor)	187		*	0.005
Netball	781		*	0.018
Rowing	80	*		0.003
Rugby union	158		*	0.024
Shooting sports	160		*	0.008
Squash/racquetball	293		*	0.045
Table tennis	112	*		0.011
Tennis	1,263		*	<0.001
Tenpin bowling	121		*	0.047

^1^Level of organisation: 1 All unorganised; 2 At least some organised by a club or other organisation; 3 At least some in a sports club or leisure centre setting requiring payment.

^2^For the remaining 75 types of physical activity, either there was no significant relationship between SEIFA IRSAD quintile and the rate of regular participation, or the sample size was too small for valid statistical analysis.

^3^p-value for Goodman and Kruskal’s gamma coefficient.

Table 6 shows the nature of the relationship between rate of any participation in PA and ARIA+ category (numbered from 1–3, with 1 being the major cities). Thirty two of the 95 types of PA were shown to have a significant relationship between rate of participation and ARIA+ category. Fifteen had a positive linear relationship between rate of participation and ARIA+ category, with seven of these also having a superimposed non-linear relationship. Eleven had a negative linear relationship between rate of participation and ARIA+ category, of which four also had a superimposed non-linear relationship. Three had a purely non-linear relationship between participation and ARIA+ category, with no significant linear trend. Additional file 1: Table S6A provides detailed examples illustrating different patterns of relationship.

**Table 6:** **Relationship between rate of participation in particular types of physical activity and ARIA+ remoteness category**

		**Relationship with ARIA+ category**			
**Physical activity** ^**1**^	**n**	**Positive linear**	**Negative linear**	**Non- linear** ^**2**^	**p-value** ^**3**^
Aerobics/fitness	21,603		*	*	<0.001
Athletics/track and field	21,604				0.038
Australian rules football	21,603	*		*	<0.001
Badminton	21,603		*		0.003
Basketball	21,604				0.022
Cricket (outdoor)	21,603	*		*	<0.001
Cycling	21,602		*	*	<0.001
Fishing	21,603	*			<0.001
Football (indoor)	21,603		*		<0.001
Golf	21,604			*	<0.001
Gridiron	21,604				0.026
Hockey (indoor)	21,603	*			0.005
Hockey (outdoor)	21,602	*		*	<0.001
Horse riding/equestrian/polo cross	21,602	*		*	<0.001
Ice/snow sports	21,604		*		0.004
Lawn bowls	21,603	*		*	<0.001
Martial arts	21,603			*	0.020
Motor sports	21,602	*		*	<0.001
Netball	21,604	*			<0.001
Rugby league	21,603	*			<0.001
Running	21,602		*	*	<0.001
Sailing	21,604			*	0.018
Scuba diving	21,603	*		*	0.001
Shooting sports	21,603	*			<0.001
Squash/racquetball	21,603	*			0.001
Surf sports	21,604		*		0.001
Swimming	21,604		*	*	<0.001
Tenpin bowling	21,603		*		0.001
Touch football	21,603	*			<0.001
Water-skiing/power boating	21,603	*			<0.001
Weight training	21,604		*		<0.001
Yoga	21,603		*	*	<0.001

^1^For the remaining 63 types of physical activity, there was no significant relationship between ARIA+ category and the rate of regular participation.

^2^Second order (quadratic) relationship.

^3^Logistic regression likelihood ratio test.

Table 7 shows the nature of the relationship between the rate of regular participation and ARIA+ category. Fifteen of the 95 types of PA were shown to have a significant relationship between rate of participation and ARIA+ category. Five had a positive linear relationship between rate of regular participation and ARIA+ category, with two of these also having a superimposed non-linear relationship. Three had a negative linear relationship between rate of participation and ARIA+ category, of which one also had a superimposed non-linear relationship. Five had a purely non-linear relationship between participation and ARIA+ category, with no significant linear trend. Additional file 1: Table S7A provides detailed examples illustrating different patterns of relationship.

**Table 7:** **Relationship between rate of regular** ^**1**^ **participation in particular types of physical activity and ARIA+ remoteness category**

		**Relationship with ARIA+ category**			
**Physical activity** ^**2**^	**n**	**Positive linear**	**Negative linear**	**Non- linear** ^**3**^	**p-value** ^**4**^
Aerobics/Fitness	5,070		*		0.001
Australian Rules Football	712			*	0.013
Basketball	747			*	<0.001
Bush Walking	1,040	*			<0.001
Cycling	2,562				0.012
Fishing	481	*			0.014
Hockey (indoor)	33			*	0.004
Ice/Snow Sports	273				0.023
Motor Sports	290			*	<0.001
Orienteering	156			*	0.001
Shooting Sports	165	*		*	0.003
Squash/Racquetball	296	*		*	0.004
Swimming	2,799	*			0.001
Table Tennis	120		*		0.046
Touch Football	597		*	*	<0.001

^1^Twelve times or more v fewer than 12 times in past 12 months.

^2^For the remaining 80 types of physical activity, either there was no significant relationship between ARIA+ category and the rate of regular participation, or the sample size was too small for valid statistical analysis.

^3^Second order (quadratic) relationship.

^4^Logistic regression likelihood ratio test.

Table 8 shows the nature of the relationship between level of organisation of participation and ARIA+ category. Seventeen of the 95 types of PA were shown to have a significant relationship (concordance) between level of organisation of PA and ARIA+ category quintile. Fourteen concordances were positive and three were negative.

**Table 8:** **Relationship between level of organisation** ^**1**^ **of physical particular types of activity and ARIA+ remoteness category**

		**Relationship with ARIA+ category**		
**Physical activity** ^**2**^	**n**	**Positive**	**Negative**	**p-value** ^**3**^
Air Sports	16	*		0.001
Australian Rules Football	687	*		<0.001
Badminton	155	*		0.014
Baseball	39		*	0.003
Basketball	746	*		<0.001
Bush Walking	991		*	0.010
Cricket (outdoor)	676	*		0.001
Football (outdoor)	1,010	*		<0.001
Golf	1,412	*		<0.001
Hockey (outdoor)	189	*		0.005
Netball	781	*		<0.001
Rugby Union	160	*		0.020
Running	2,175	*		<0.001
Squash/Racquetball	295	*		0.005
Tennis	1,264	*		<0.001
Touch Football	569	*		0.001
Yoga	662		*	<0.001

^1^Level of organisation: All unorganised; At least some organised by a club or other organisation; At least some in a sports club or leisure centre setting requiring payment.

^2^For the remaining 78 types of physical activity, either there was no significant relationship between ARIA+ category and the rate of regular participation, or the sample size was too small for valid statistical analysis.

^3^p-value for Goodman and Kruskal’s gamma coefficient.

## Discussion

This study provides detailed information about the associations between participation in particular sports and physical activities and measures of SES and location. It demonstrates the complexity of these associations across different contexts of participation.

### SES

Many studies have shown a broad association between higher SES and higher levels of PA and sport [5,6,20,21] and the present study confirms this positive overall association, both for any recreational PA participation in a 12-month period and for regular participation in some form of PA over that period. However, more specifically this study demonstrates that only 42 (44%) of the 95 specific types of PA showed a significant association between participation and neighbourhood SES. Furthermore, in even fewer cases (n = 25; 26%) was the association positive, with high/low participation being associated with high/low SES.

SES can be defined in terms of individual, household and neighbourhood characteristics. The socioeconomic inequalities in sport participation have been explained by a combination of individual, household and neighbourhood factors [20]. Lower PA levels have been associated with lower neighbourhood and household SES (education, income) [5]. Participation in club sport by adolescent females has been significantly positively associated with neighbourhood and household measures of SES, particularly in metropolitan compared to regional/rural areas [7].

In the present study, for each of 95 different types of sport or PA, a neighbourhood SES measure was associated with rate of participation, rate of regular participation and level of organisation of the context of participation. Significant associations were observed between SES and a minority of activities - for any participation (42 activities), regular participation (21 activities) and participation in organised contexts (20 activities).

There were relatively few (n = 25) activities for which the rate of any participation increased as SES increased. For only two activities (Athletics/Track and Field, and Basketball) did the rate of regular participation increase as SES increased. For six activities, the proportion participating in more organised contexts increased as SES increased. For all three aspects of participation, the positive associations between participation and SES generally occurred for ‘niche’ sports and activities (such as canoeing/kayaking, rock climbing, rowing) rather than the more popular ‘mainstream’ sports (such as cricket, netball).

There were similar minorities of sports exhibiting negative relationships between the three aspects of participation (any, regular, level of organisation) and SES. Of the activities showing a negative relationship between any participation and SES, the majority were team sports. Negative relationships between participation in more organised contexts and SES were also more likely to exist for team sports, such as Australian rules football, basketball, football, hockey, netball and tennis.

From the numbers of sports listed in Tables 3,4,5, it would seem that SES is a significant correlate of participation in only a minority of sports, and is more likely to be associated with participation in general rather than for regular participation or participation in more organised contexts. Further, more complex non-linear relationships predominate over clear positive and negative trends. This contrasts somewhat with the positive overall association observed in this study, and in other studies that have reported significantly higher rates of PA in general, and organised sport participation in particular, for higher SES compared to lower SES [6,21]. Clearly, the general positive relationship between SES and participation does not apply uniformly to all types of sport and PA..

Further examination suggests that types of PA which: are undertaken indoors; are likely to require expensive infrastructure or equipment; or require access to water or snow, were more likely to exhibit positive relationships between participation and SES. Indoor activities such as yoga often require a fee for each participation session, in contrast to many club sports which have a yearly membership rather than an individual pay-and-play system. The cost of equipment is often a determinant of participation [6,22]. It is a common finding that people of higher SES have better access to PA and sports facilities, can afford to live in a PA-friendly environment and have fewer barriers [23].

Studies investigating broad levels of PA have reported that access to low-cost recreation facilities can significantly, positively influence PA levels [24]. Recent research in Spain found that the odds for prevalence of physical activity were lower in neighbourhoods of lower income [25]. The availability of sports facilities explained much of the excess prevalence in older years, but not for younger people [25]. Other studies have reported fewer facilities within lower SES compared to higher SES neighbourhoods, indicating that the physical environment hinders the ability in the lower SES categories to access PA opportunities [9]. Furthermore, the access to low-cost recreation facilities is not consistent and quite variable between countries. In a comparison of 11 countries, availability of low-cost recreation facilities was least likely to be reported in Brazil and Columbia and most likely in Canada and New Zealand [24].

Notwithstanding the above, for some activities that can have very low participation costs (such as running and cycling) participation was positively associated with SES, although not in a clearly linear fashion. Whilst cycling can be an expensive activity in terms of equipment, running does not incur expenses above and beyond shoes. We know that people with higher SES are likely to have higher education, and it is reported that people with higher education have amongst other things, more social support and greater capacity to seek, understand and act on health messages that promote PA [23]. It may be that activities such as cycling and running provide easy options that do not require skills, facilities nor other people to participate with. It may also be that people from higher SES neighbourhoods have a more aesthetic environment which is more conducive to running and/or cycling, or they may feel safer. Conversely, poor health, cost, unfamiliarity of PA facilities and programs, limited social support and living in an unsafe neighbourhood are barriers to men from low SES being physically active [26].

From an equity perspective, it is a positive finding that rates of participation in many physical activities are not positively associated with levels of SES. For some activities, participation decreased as SES increased. These were predominantly organised team sports such as Australian rules football, basketball, cricket, hockey, netball and tennis. We can conclude that many traditional Australian team sports are either not associated with SES in a prohibitive manner, or in some cases are more likely to be participated in by people from lower SES areas.

### ARIA

We found that the rates of both PA participation in general and regular PA participation decreased as remoteness (ARIA+ category) increased, and the level of organisation of PA context increased as remoteness increased. However, for specific activities, significant associations between participation levels and remoteness occurred in only a minority of the 95 cases.

Significant associations were observed between remoteness and participation in general for 32 activities. However, for only 11 activities did the rate of participation decrease with increasing remoteness. For 15 activities, the rate of participation was higher in more remote areas; furthermore, these included some of the most popular mainstream sports – Australian rules football, cricket, netball, hockey and lawn bowls, as well as typical rural PA pursuits such as fishing [27]. The activities for which participation rates declined with increasing remoteness included a number requiring indoor facilities – aerobics/fitness, indoor football, tenpin bowling, weight training and yoga, consistent with the notion that infrastructure differences between metropolitan and rural settings can have an effect on participation [10].

For only a small proportion of activities (n = 15) was remoteness associated with the rate of regular participation, and the direction and shape of these relationships was mixed. There was however a much more consistent pattern with regard to level of organisation, with more organised participation in more remote areas, again including some of the most popular mainstream sports – Australian rules football, basketball, cricket, football, netball, hockey and tennis. This suggests that for these activities, sporting clubs and organisations tend to play a more important role in rural than metropolitan communities.

The limited research discussing differences across geographical locations suggests that in rural communities there is likely to be an emphasis on traditional team sports and more limited choices than those available in metropolitan areas [12]. Nevertheless, a study of younger people (9–16 years) found that overall, time participating in organised sport did not differ for those living in major cities compared to regional and remote residents [14]. However, recent research with adults has reported that PA levels are lower in regional communities than state averages [11]. This study also found that there were different PA patterns in different regional communities. The proportion of people reporting no activity was higher in some regions than others, which the authors suggested may be due to infrastructure for activity, as well as workplace policies and programs [11]. Another factor suggested by the respondents in this study is that their rural work and lifestyles required a considerable amount of PA already [11].

In summary, it is encouraging that participation in many traditional Australian team sports was not found to be positively associated with SES nor negatively associated with remoteness. Team sports are social in nature, and we know that people are inherently motivated to participate in sport for social opportunities [8,11]. Team sport participation, in addition to producing physical health benefits can enhance psychological and social health [28]. A study across three different countries found that sport delivery systems that create social opportunities may be a key to increased adult sport participation [8]. The social context of sport has also been identified as a mechanism for assisting men of low SES to overcome isolation [26]. These authors advocated the use of sport as a vehicle to achieve social inclusion.

### Strengths and Limitations

A strength of this study is that it is based on a very large national dataset. This is a double edged sword however, in that the resulting high statistical power may result in statistical significance in cases where the strength of the association is insufficient to be of great practical importance. Also, because of the large number of significance tests conducted, it is acknowledged that some of the relationships identified as significant will be spurious and due to Type 1 errors, i.e. chance patterns of participation within the ERASS survey sample. However, the rate of results significant at the 0.05 level in each table far exceeds the chance rate of one in 20, indicating that most of the significant results reported are valid and meaningful.

Another methodological limitation is that, because the ERASS survey did not include questions about individual or household SES, the measure of SES used was based on postal area. Further, ERASS data are limited to persons aged 15 years or more. The patterns of relationship between participation, SES and remoteness may be very different for children younger than 15 years.

## Conclusions

In conclusion, it is encouraging that few types of PA were cost- or remoteness-prohibitive in terms of participation. As remoteness increased and SES decreased, participation in many team sports actually increased. For both SES and remoteness, there were more significant associations with overall participation, than with regular participation or participation in more organised contexts. This suggests that once initial engagement in PA is established, SES and remoteness are not critical determinants of the depth of engagement. Furthermore, it would seem inappropriate to generalise regarding SES and location. The level of contextual differentiation means that policies to promote PA participation based on generalisations may be poorly targeted. It is important that programs and policies designed to increase participation in PA take into account the strong contextual factors.

## Additional file

Additional file 1:
**Table S1.** List of 95 designated ERASS physical activity types. **Table S3A.** Examples of different patterns of relationship between rate of participation in particular types of physical activity and quintiles of SEIFA IRSAD. **Table S4A.** Examples of different patterns of relationship between rate of regular participation in particular types of physical activity and quintiles of SEIFA IRSAD. **Table S6A.** Examples of different patterns of relationship between rate of participation in particular types of physical activity and ARIA+ remoteness category. **Table S7A.** Examples of different patterns of relationship between regular participation in particular types of physical activity and ARIA+ remoteness category.

## Abbreviations

ABS: Australian Bureau of Statistics
ARIA: Accessibility/Remoteness Index of Australia
ERASS: Exercise Recreation and Sport Survey
IRSAD: Index of Relative Socio-economic Advantage and Disadvantage
PA: Physical Activity
SEIFA: Socio-economic Indexes for Areas
SES: socio-economic status
