# Supplementary material for: Participation in sport and physical activity: associations with socio-economic status and geographical remoteness
Source: BMC Public Health. 2015 Apr 29;15:434. doi: 10.1186/s12889-015-1796-0 (PMC4423100; doi:10.1186/s12889-015-1796-0)
Supplement: Additional file 1: — Table S1. List of 95 designated ERASS physical activity types. Table S3A. Examples of different patterns of relationship between rate of participation in particular types of physical activity and quintiles of SEIFA IRSAD. Table S4A. Examples of different patterns of relationship between rate of regular participation in particular types of physical activity and quintiles of SEIFA IRSAD. Table S6A. Examples of different patterns of relationship between rate of participation in particular types of physical activity and ARIA+ remoteness category. Table S7A. Examples of different patterns of relationship between regular participation in particular types of physical activity and ARIA+ remoteness category. [file 12889_2015_1796_MOESM1_ESM.docx]

Supplementary Table 1. List of 95 designated ERASS physical activity types

| Aerobics/Fitness | Golf | Running |
| --- | --- | --- |
| Air Sports | Gorilla Ball | Sailing |
| Aquarobics | Gridiron | Scuba Diving |
| Archery/Bow Hunting | Grockey | Sheepdog Trails |
| Athletics/Track and Field | Gymnastics | Shooting Sports |
| Australian Rules Football | Handball | Soft Crosse |
| Badminton | Hockey (indoor) | Softball |
| Baseball | Hockey (outdoor) | Squash/Racquetball |
| Basketball | Horse Racing | Surf Lifesaving |
| Billiards/Snooker/Pool | Horse Riding/Equestrian/Polo Cross | Surf Sports |
| Bocce | Ice/Snow Sports | Swimming |
| Boomerang Throwing | Korfball | Table Tennis |
| Boxing | Lacrosse (indoor) | Tee Ball |
| Broom Ball | Lacrosse (outdoor) | Tennis |
| Bush Walking | Lawn Bowls | Tenpin Bowling |
| Canoe Polo | Leader Ball | Touch Football |
| Canoeing/Kayaking | Marching | Triathlons |
| Carpet Bowls | Martial Arts | Ultimate Frisbee |
| Commonwealth Games | Motor Sports | Underwater Hockey |
| Cricket (indoor) | Netball | Volleyball |
| Cricket (outdoor) | Orienteering | Walking |
| Croquet | Other Activities | Water Polo |
| Cycling | Pigeon Racing | Water Volleyball |
| Dancing | Play | Water-skiing/Power Boating |
| Darts | Putt Putt Golf | Weight Lifting Competition |
| Dog Racing | Rock Climbing | Weight Training |
| Electric Light Cricket | Rodeo | Wheelchair Ice Hockey |
| Fencing | Roller Sports | Winter Olympics |
| Fishing | Rowing | Wood Chopping |
| Football (indoor) | Royal Tennis | Wrestling |
| Football (outdoor) | Rugby League | Yoga |
| Gaelic Football | Rugby Union |  |

Supplementary Table 3A. Examples of different patterns of relationship between rate of participation in particular types of physical activity and quintiles of SEIFA IRSAD

|  | Did not participate | | Did participate | |  |  |  |
| --- | --- | --- | --- | --- | --- | --- | --- |
| SEIFA IRSAD quintile | n | % | n | % | OR | (95% CI) | p-value |
| Walking^1^ | 13849 | 96.7 | 7,749 | 3.3 |  |  |  |
| 1 (619.55-938.8) | 2614 | 67.2 | 1,276 | 32.8 | 1.00 |  |  |
| 2 (938.81-979.77) | 2933 | 66.6 | 1,472 | 33.4 | 0.94 | (0.94, 1.13) | 0.554 |
| 3 (979.79-1019.46) | 2793 | 63.3 | 1,619 | 36.7 | 1.38 | (1.08, 1.3) | <0.001 |
| 4 (1019.48-1065.35) | 2703 | 63.2 | 1,575 | 36.8 | 1.78 | (1.09, 1.31) | <0.001 |
| 5 (1065.51-1164.41) | 2806 | 60.8 | 1,807 | 39.2 | 2.29 | (1.21, 1.44) | <0.001 |
|  |  |  |  |  |  |  |  |
| Lawn Bowls^2^ | 21154 | 97.9 | 444 | 2.1 |  |  |  |
| 1 (619.55-938.8) | 3778 | 97.1 | 112 | 2.9 | 1.00 |  |  |
| 2 (938.81-979.77) | 4292 | 97.4 | 113 | 2.6 | 0.89 | (0.68, 1.16) | 0.381 |
| 3 (979.79-1019.46) | 4323 | 98.0 | 89 | 2.0 | 0.69 | (0.52, 0.92) | 0.011 |
| 4 (1019.48-1065.35) | 4214 | 98.5 | 64 | 1.5 | 0.51 | (0.37, 0.7) | <0.001 |
| 5 (1065.51-1164.41) | 4547 | 98.6 | 66 | 1.4 | 0.49 | (0.36, 0.66) | <0.001 |
|  |  |  |  |  |  |  |  |
| Aerobics/Fitness^3^ | 16517 | 76.5 | 5,080 | 23.5 |  |  |  |
| 1 (619.55-938.8) | 3160 | 81.2 | 730 | 18.8 | 1.00 |  |  |
| 2 (938.81-979.77) | 3533 | 80.2 | 873 | 19.8 | 1.07 | (0.96, 1.19) | 0.225 |
| 3 (979.79-1019.46) | 3416 | 77.4 | 995 | 22.6 | 1.26 | (1.13, 1.4) | <0.001 |
| 4 (1019.48-1065.35) | 3192 | 74.6 | 1,086 | 25.4 | 1.47 | (1.33, 1.64) | <0.001 |
| 5 (1065.51-1164.41) | 3216 | 69.7 | 1,396 | 30.3 | 1.88 | (1.7, 2.08) | <0.001 |
|  |  |  |  |  |  |  |  |
| Australian Rules Football^4^ | 20886 | 96.7 | 712 | 3.3 |  |  |  |
| 1 (619.55-938.8) | 3793 | 97.5 | 97 | 2.5 | 1.00 |  |  |
| 2 (938.81-979.77) | 4261 | 96.7 | 144 | 3.3 | 1.32 | (1.02, 1.71) | 0.037 |
| 3 (979.79-1019.46) | 4211 | 95.4 | 201 | 4.6 | 1.86 | (1.45, 2.38) | <0.001 |
| 4 (1019.48-1065.35) | 4130 | 96.5 | 149 | 3.5 | 1.40 | (1.08, 1.81) | 0.011 |
| 5 (1065.51-1164.41) | 4491 | 97.4 | 121 | 2.6 | 1.05 | (0.8, 1.38) | 0.714 |

1 Positive linear relationship.

2 Negative linear relationship.

3 Positive linear trend with additional non-linear component.

4 No linear trend: non-linear relationship only.

Supplementary Table 4A. Examples of different patterns of relationship between rate of regular participation in particular types of physical activity and quintiles of SEIFA IRSAD

|  | Participated <12 times | | Participated ≥12 times | |  |  |  |
| --- | --- | --- | --- | --- | --- | --- | --- |
| SEIFA IRSAD Quintile | n | % | n | % | OR | (95% CI) | p-value |
| Athletics/Track & Field^1^ | 44 | 30.6 | 100 | 69.4 |  |  |  |
| 1 (619.55-938.8) | 25 | 65.8 | 13 | 34.2 | 1.00 |  |  |
| 2 (938.81-979.77) | 6 | 26.1 | 17 | 73.9 | 5.66 | (1.79,17.89) | 0.003 |
| 3 (979.79-1019.46) | 7 | 25.0 | 21 | 75.0 | 5.54 | (1.88,16.28) | 0.002 |
| 4 (1019.48-1065.35) | 2 | 8.0 | 23 | 92.0 | 27.21 | (4.81,153.86) | <0.001 |
| 5 (1065.51-1164.41) | 4 | 13.3 | 26 | 86.7 | 12.60 | (3.62,43.83) | <0.001 |
|  |  |  |  |  |  |  |  |
| Water Skiing/Power Boating^2^ | 53 | 37.3 | 89 | 62.7 |  |  |  |
| 1 (619.55-938.8) | 6 | 27.3 | 16 | 72.7 | 1.00 |  |  |
| 2 (938.81-979.77) | 9 | 21.4 | 33 | 78.6 | 1.50 | (0.46,4.86) | 0.497 |
| 3 (979.79-1019.46) | 10 | 37.0 | 17 | 63.0 | 0.71 | (0.21,2.35) | 0.570 |
| 4 (1019.48-1065.35) | 17 | 54.8 | 14 | 45.2 | 0.32 | (0.10,1.03) | 0.057 |
| 5 (1065.51-1164.41) | 11 | 55.0 | 9 | 45.0 | 0.33 | (0.09,1.16) | 0.085 |
|  |  |  |  |  |  |  |  |
| Shooting Sports^3^ | 63 | 38.4 | 101 | 61.6 |  |  |  |
| 1 (619.55-938.8) | 12 | 21.8 | 43 | 78.2 | 1.00 |  |  |
| 2 (938.81-979.77) | 11 | 39.3 | 17 | 60.7 | 0.42 | (0.16,1.14) | 0.089 |
| 3 (979.79-1019.46) | 1 | 4.5 | 21 | 95.5 | 3.88 | (0.65,23.30) | 0.138 |
| 4 (1019.48-1065.35) | 22 | 66.7 | 11 | 33.3 | 0.15 | (0.06,0.38) | <0.001 |
| 5 (1065.51-1164.41) | 17 | 65.4 | 9 | 34.6 | 0.15 | (0.05,0.41) | <0.001 |
|  |  |  |  |  |  |  |  |
| Football (outdoor)^4^ | 225 | 21.7 | 811 | 78.3 |  |  |  |
| 1 (619.55-938.8) | 26 | 21.1 | 97 | 78.9 | 1 |  |  |
| 2 (938.81-979.77) | 43 | 15.8 | 229 | 84.2 | 1.45 | (0.84,2.48) | 0.181 |
| 3 (979.79-1019.46) | 65 | 30.1 | 151 | 69.9 | 0.63 | (0.37,1.06) | 0.082 |
| 4 (1019.48-1065.35) | 32 | 15.1 | 180 | 84.9 | 1.52 | (0.86,2.7) | 0.150 |
| 5 (1065.51-1164.41) | 59 | 27.7 | 154 | 72.3 | 0.71 | (0.42,1.2) | 0.196 |

^1^ Positive linear relationship.

^2^ Negative linear relationship.

^3^ Negative linear trend with additional non-linear component.

^4^ No linear trend: non-linear relationship only.

Supplementary Table 6A. Examples of different patterns of relationship between rate of participation in particular types of physical activity and ARIA+ remoteness category

|  | Did not participate | | Did participate | |  |  |  |
| --- | --- | --- | --- | --- | --- | --- | --- |
| ARIA+ category | n | % | n | % | OR | (95% CI) | p-value |
| Netball^1^ | 20802 | 96.3 | 802 | 3.7 |  |  |  |
| Major City | 13984 | 96.5 | 501 | 3.5 | 1 |  |  |
| Inner Regional | 4227 | 96.3 | 161 | 3.7 | 1.06 | (0.89,1.28) | 0.498 |
| Other | 2591 | 94.9 | 140 | 5.1 | 1.50 | (1.24,1.82) | <0.001 |
|  |  |  |  |  |  |  |  |
| Weight Training^2^ | 20968 | 97.1 | 636 | 2.9 |  |  |  |
| Major City | 14010 | 96.7 | 475 | 3.3 | 1 |  |  |
| Inner Regional | 4286 | 97.7 | 103 | 2.3 | 0.71 | (0.57,0.88) | 0.002 |
| Other | 2672 | 97.9 | 58 | 2.1 | 0.64 | (0.49,0.85) | 0.002 |
|  |  |  |  |  |  |  |  |
| Australian Rules Football^3^ | 20890 | 96.7 | 713 | 3.3 |  |  |  |
| Major City | 14075 | 97.2 | 410 | 2.8 | 1 |  |  |
| Inner Regional | 4193 | 95.6 | 195 | 4.4 | 1.60 | (1.34,1.90) | <0.001 |
| Other | 2622 | 96.0 | 108 | 4.0 | 1.41 | (1.14,1.76) | 0.004 |
|  |  |  |  |  |  |  |  |
| Golf^4^ | 20151 | 93.3 | 1,453 | 6.7 |  |  |  |
| Major City | 13574 | 93.7 | 911 | 6.3 | 1 |  |  |
| Inner Regional | 4039 | 92.0 | 350 | 8.0 | 1.29 | (1.13,1.47) | <0.001 |
| Other | 2538 | 93.0 | 192 | 7.0 | 1.13 | (0.96,1.32) | 0.148 |

^1^ Positive linear relationship.

^2^ Negative linear relationship.

^3^ Positive linear trend with additional non-linear component.

^4^ No linear trend: non-linear relationship only.

Supplementary Table 7A. Examples of different patterns of relationship between regular participation in particular types of physical activity and ARIA+ remoteness category

|  | Participated | | Participated | |  |  |  |
| --- | --- | --- | --- | --- | --- | --- | --- |
|  | <12 times | | ≥12 times | |  |  |  |
| ARIA+ category | n | % | n | % | OR | (95% CI) | p |
| Swimming^1^ | 520 | 18.6 | 2,279 | 81.4 |  |  |  |
| Major City | 405 | 20.3 | 1,592 | 79.7 | 1.00 |  |  |
| Inner Regional | 74 | 15.3 | 409 | 84.7 | 1.40 | (1.07,1.83) | 0.015 |
| Other | 41 | 12.9 | 278 | 87.1 | 1.71 | (1.21,2.41) | 0.002 |
|  |  |  |  |  |  |  |  |
| Aerobics/Fitness^2^ | 277 | 5.5 | 4,793 | 94.5 |  |  |  |
| Major City | 181 | 4.8 | 3,604 | 95.2 | 1.00 |  |  |
| Inner Regional | 57 | 7.1 | 745 | 92.9 | 0.66 | (0.48,0.9) | 0.008 |
| Other | 39 | 8.1 | 444 | 91.9 | 0.57 | (0.4,0.81) | 0.002 |
|  |  |  |  |  |  |  |  |
| Touch Football^3^ | 116 | 19.4 | 481 | 80.6 |  |  |  |
| Major City | 56 | 17.9 | 256 | 82.1 | 1.00 |  |  |
| Inner Regional | 17 | 11.3 | 134 | 88.7 | 1.72 | (0.96,3.08) | 0.067 |
| Other | 43 | 32.1 | 91 | 67.9 | 0.46 | (0.29,0.74) | 0.001 |
|  |  |  |  |  |  |  |  |
| Basketball^4^ | 132 | 17.7 | 615 | 82.5 |  |  |  |
| Major City | 82 | 15.4 | 452 | 84.6 | 1.00 |  |  |
| Inner Regional | 42 | 33.1 | 85 | 66.9 | 0.36 | (0.23,0.56) | <0.001 |
| Other | 8 | 9.3 | 78 | 90.7 | 1.78 | (0.82,3.83) | 0.143 |

^1^ Positive linear relationship.

^2^ Negative linear relationship.

^3^ Positive linear trend with additional non-linear component.

^4^ No linear trend: non-linear relationship only.
